# Exploring the Progress of Hyaluronic Acid Hydrogels: Synthesis, Characteristics, and Wide-Ranging Applications

**DOI:** 10.3390/ma17102439

**Published:** 2024-05-18

**Authors:** Iman Gholamali, Trung Thang Vu, Sung-Han Jo, Sang-Hyug Park, Kwon Taek Lim

**Affiliations:** 1 Industry 4.0 Convergence Bionics Engineering, Pukyong National University, Busan 48513, Republic of Korea; imangholamali1212@pknu.ac.kr (I.G.); josunghan91@gmail.com (S.-H.J.); 2 Department of Smart Green Technology Engineering, Pukyong National University, Busan 48513, Republic of Korea; vutrungthang29@gmail.com; 3 Major of Biomedical Engineering, Division of Smart Healthcare, College of Information Technology and Convergence, Pukyong National University, Busan 48513, Republic of Korea; 4 Institute of Display Semiconductor Technology, Pukyong National University, Busan 48513, Republic of Korea

**Keywords:** hyaluronic acid, hydrogel, extracellular matrix, biomedicine, cross-linking technique

## Abstract

This comprehensive review delves into the world of hyaluronic acid (HA) hydrogels, exploring their creation, characteristics, research methodologies, and uses. HA hydrogels stand out among natural polysaccharides due to their distinct features. Their exceptional biocompatibility makes them a top choice for diverse biomedical purposes, with a great ability to coexist harmoniously with living cells and tissues. Furthermore, their biodegradability permits their gradual breakdown by bodily enzymes, enabling the creation of temporary frameworks for tissue engineering endeavors. Additionally, since HA is a vital component of the extracellular matrix (ECM) in numerous tissues, HA hydrogels can replicate the ECM’s structure and functions. This mimicry is pivotal in tissue engineering applications by providing an ideal setting for cellular growth and maturation. Various cross-linking techniques like chemical, physical, enzymatic, and hybrid methods impact the mechanical strength, swelling capacity, and degradation speed of the hydrogels. Assessment tools such as rheological analysis, electron microscopy, spectroscopy, swelling tests, and degradation studies are employed to examine their attributes. HA-based hydrogels feature prominently in tissue engineering, drug distribution, wound recovery, ophthalmology, and cartilage mending. Crafting HA hydrogels enables the production of biomaterials with sought-after qualities, offering avenues for advancements in the realm of biomedicine.

## 1. Introduction

A hydrogel is a gel-like material consisting of interlinked hydrophilic polymer chains that exhibit exceptional water absorption and retention capabilities. When hydrated, it swells and forms a three-dimensional network, showcasing its distinctive properties [1]. Hydrogels can be derived from either natural or synthetic polymers, and their physical and chemical attributes can differ depending on the precise composition and cross-linking of the polymer chains [2,3]. Capitalizing on their biocompatible nature, soft and flexible characteristics, and ability to replicate certain properties of biological tissues, hydrogels have gained extensive utilization across various domains [4,5].

Natural polymer hydrogels, such as gelatin, collagen, alginate, chitosan, hyaluronic acid (HA), and cellulose, undergo cross-linking through physical or chemical interactions, leading to the formation of hydrogels with a wide range of mechanical, chemical, and biological properties [6,7]. These hydrogels find extensive application in various biomedical fields, including drug delivery systems [8], tissue engineering [9], wound healing [10], scaffolds for promoting cell growth [11], and tissue regeneration [12]. Their inherent biocompatibility and similarity to the extracellular environment have generated significant interest in research and development activities focused on medical and pharmaceutical hydrogel applications [13].

The HA hydrogel, primarily comprised of endogenous HA, embodies a gel-like structure [14]. HA hydrogels can be synthesized using different methods, including chemical cross-linking, physical cross-linking, and enzymatic cross-linking. Chemical cross-linking involves using chemical agents like glutaraldehyde, carbodiimides, divinyl sulfone, dimaleimide, etc., to create covalent bonds between HA molecules. Physical cross-linking, on the other hand, uses physical stimuli such as temperature, pH, and ionic strength to induce gelation of HA. Enzymatic cross-linking uses enzymes like transglutaminase to form covalent bonds between HA molecules [6,9]. The synthesis method chosen can affect the mechanical properties, swelling behavior, and degradation rate of the resulting hydrogel. Additionally, the characteristics of HA hydrogels, such as their molecular weight, degree of cross-linking, and concentration, can also influence their properties and applications. Higher molecular weight HA hydrogels tend to have higher viscosity and slower degradation rates, while lower molecular weight HA hydrogels tend to have lower viscosity and faster degradation rates [9,14]. Remarkably versatile, they boast widespread utilization across diverse biomedical domains encompassing drug delivery systems, tissue engineering, wound healing, and scaffolds to support and foster cellular growth and regeneration [15,16,17]. Notably, HA hydrogels have garnered substantial research interest within the field of regenerative medicine, owing to their remarkable capability to mimic the intricacies of the extracellular matrix (ECM), incite cell proliferation, and promote the regeneration of tissues [18,19]. Overall, the synthesis and characteristics of HA hydrogels are crucial in determining their properties and potential applications in tissue engineering and regenerative medicine.

This review article serves as a valuable resource for researchers and scientists to stay up-to-date with the latest advancements in their field. It can offer a broad perspective on new synthesis methods, characterization techniques, and emerging applications. Additionally, it can provide a thorough understanding of the properties and characteristics of HA hydrogels, which can aid in exploring their potential applications. By identifying areas that require further research, the review article can facilitate progress in the field and encourage innovation.

## 2. Synthesis of HA Hydrogels

HA is a fascinating and versatile macromolecule found in nature, intricately intertwined in the ECM. Known for its natural compatibility, biodegradability, and non-immunogenic properties, HA is an ideal building block for creating hydrogels for various biomedical purposes. The extensive network of HA bulk hydrogels serves as excellent scaffolds for applications like tissue engineering, promoting cell infiltration, and nutrient diffusion crucial for activities like skin and cartilage regeneration [20]. On the other hand, microscopic HA hydrogel particles, such as HGPs, microgels, and nanogels, are perfect for targeted drug and gene delivery as they can encapsulate therapeutic agents and guide them to specific tissues or cells. The choice of synthesis method impacts the characteristics and utility of the resulting hydrogels. Chemical cross-linking offers adjustable mechanical strength and degradation properties but involves complex steps and chemicals. Physical gelation, while simpler, produces fewer durable gels. For injectable HA hydrogel particles, techniques like click chemistry, such as Diels–Alder reactions, are commonly used to create cross-linked networks rapidly at body temperatures. Enzymatic cross-linking ensures excellent biocompatibility but may introduce variability in gelation kinetics across batches. The selection of the synthesis approach depends on factors like the intended scale of application, release profile, and stability requirements in vivo. Further details on synthesis techniques are elaborated in the subsequent sections [21]. Table 1 provides a comparative summary of various chemical, physical, and enzymatic cross-linking methods that have been utilized for forming HA hydrogels, listing the specific reagents/conditions used, representative applications, and relevant references for each method.

### 2.1. Chemical Cross-Linking

Chemical cross-linking is a method used to create stable three-dimensional networks in HA hydrogels. It involves the formation of covalent bonds between HA chains [47]. By cross-linking HA, the mechanical properties and stability of the hydrogel are improved, making it suitable for a wide range of biomedical applications [48]. There are several commonly used methods for the chemical cross-linking of HA hydrogels.

#### 2.1.1. Carbodiimide Cross-Linking

**Figure 1:**
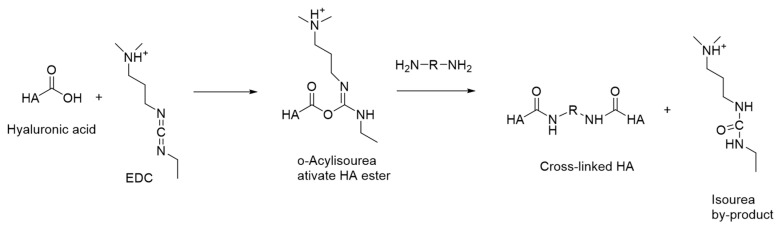
Exploring the reaction mechanism between HA and EDC.

A carbodiimide compound such as 1-ethyl-3-(3-dimethylaminopropyl) carbodiimide (EDC) is a widely used activating agent for cross-linking HA, which is often employed to establish stable covalent bonds between carboxyl groups found in biopolymers and primary amines. To initiate the reaction, the biomolecules intended for cross-linking are dissolved in a buffer solution with a slightly acidic pH (around pH 5–6) [49,50]. The reaction mixture is then supplemented with EDC, which reacts with the carboxyl groups in the biopolymers to form an activated intermediate. For the cross-link to occur, the active intermediate (such as O-acylisourea) is displaced via a nucleophilic attack from primary amines (-NH_2_) of cross-linkers to form amide bonds [22]. The reaction mixture is incubated at room temperature or a slightly elevated temperature for a specific duration, allowing the cross-linking reaction to take place. Once the cross-linking reaction is complete, purification steps such as dialysis, chromatography, or precipitation are typically performed on the resulting cross-linked biomolecules or complexes to remove excess reagents and unreacted biomolecules. The observed phenomenon can be elucidated by examining the reaction mechanism between the carboxyl groups of HA and amine groups of diamines through carbodiimide conjugation, as illustrated in Figure 1.

#### 2.1.2. Diisocyanate Cross-Linking

**Figure 2:**
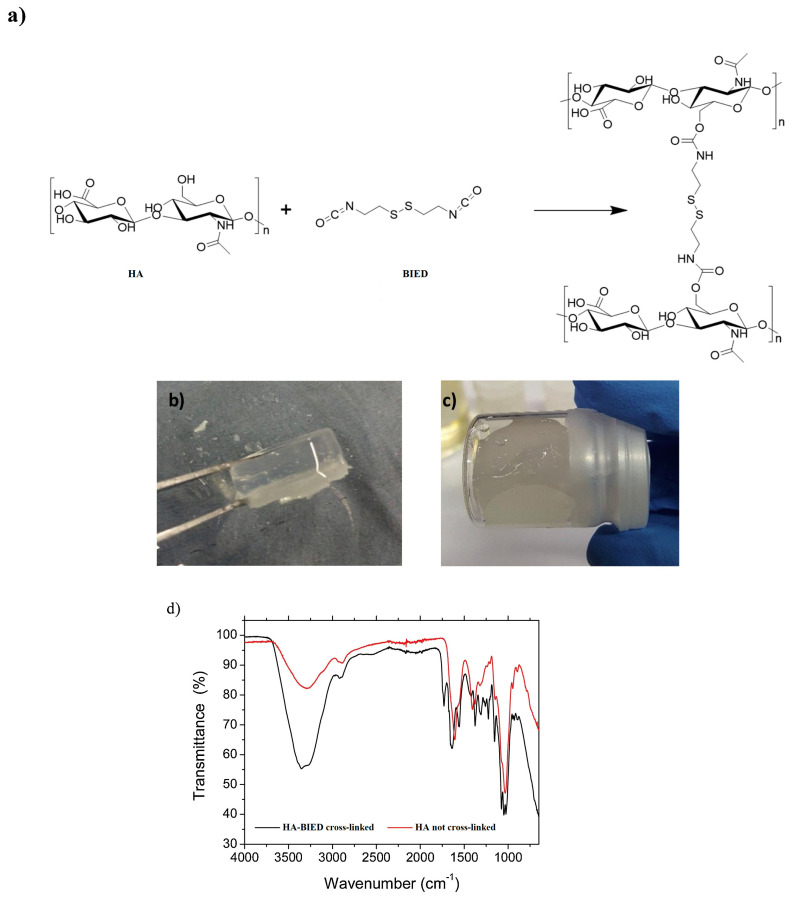
Schematic reaction between HA and BIED and a representative image of BIED-cross-linked HA gels with MW of 1.2 MDa. (**a**) Cross-linked HA demonstrates the formation of urethane bonds between the isocyanate and hydroxyl groups. (**b**) Formulation four maintains its shape. (**c**) Formulation three shows inadequate structural stability. (**d**) Representative FTIR spectra of HA and HA-BIED-cross-linked gel displaying characteristic urethane bridges. Reprinted with permission from ref. [52]. 2020, Elsevier.

The method of diisocyanate cross-linking HA involves the use of diisocyanate compounds to create a hydrogel network [49] (Figure 2). This method is commonly employed in wound healing and drug delivery applications due to the exceptional biocompatibility and biodegradability of HA [51].

Here is a step-by-step breakdown of the procedure:

HA solution preparation: A solution of HA is prepared by dissolving it in a solvent such as water or an aqueous buffer. The concentration of HA can be adjusted according to desired hydrogel properties [52].

Addition of diisocyanate cross-linker: A diisocyanate compound, such as hexamethylene diisocyanate (HDI) [23] is added to the HA solution. The diisocyanate functions as a cross-linker, forming covalent bonds between the HA chains [52]. The HA solution and diisocyanate cross-linker are thoroughly mixed to achieve uniform distribution. The cross-linking reaction occurs between the diisocyanate functional groups and the hydroxyl groups present in HA molecules.

Gelation and solidification: As the cross-linking reaction progresses, the HA solution gradually undergoes gelation, transforming into a hydrogel structure. The gelation time can be controlled by factors such as HA concentration, cross-linker concentration, and reaction conditions [52].

Characterization and application: The resulting diisocyanate-cross-linked HA hydrogel can be characterized using techniques such as rheological analysis, swelling studies, and mechanical testing. It can then be utilized for various applications, including tissue engineering scaffolds, drug delivery systems, and regenerative medicine [52,53].

#### 2.1.3. Michael Addition

**Figure 3:**
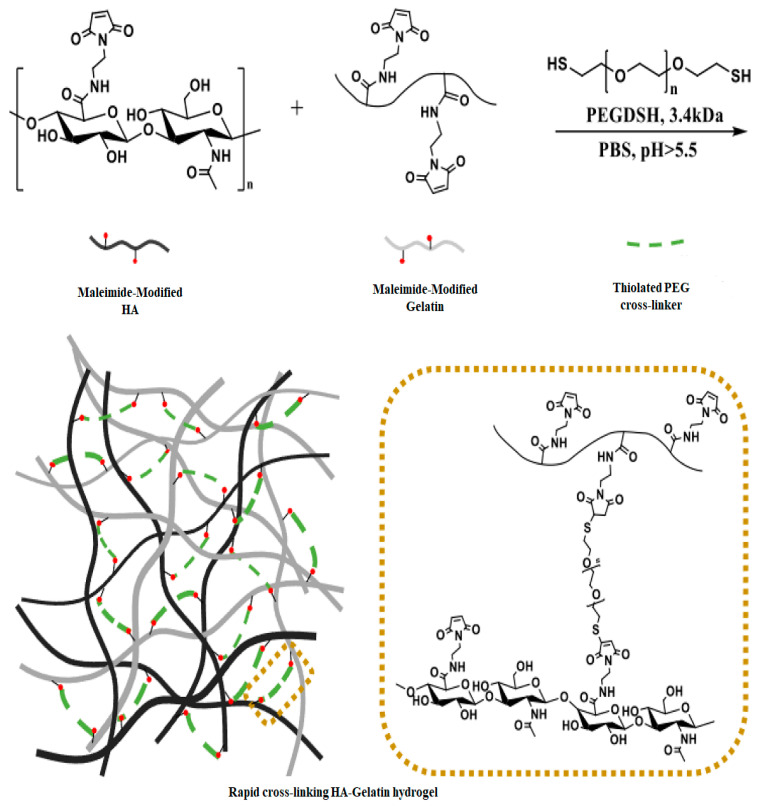
The hydrogel formulation was depicted through schematic representations, illustrating the swift cross-linking reaction with HA-Mal, Gel-Mal, and PEGDSH in PBS. Reprinted with permission from ref. [27]. 2021, MDPI.

The Michael addition method is a technique used to produce hydrogels through a chemical reaction called the Michael addition. This method involves linking HA molecules together to create a three-dimensional network, resulting in the formation of a hydrogel with desirable properties [24,54]. In this process, cross-linkers containing thiol groups, such as cysteine or dithiothreitol (DTT) [25,26], react with HA modified with acrylate groups. The reaction occurs between the thiol functional groups of HA and another reactive molecule, typically a cross-linker with vinyl sulfone (-VS) [55], maleimide (-MAL) [27], or acrylate (-AC) [28] reactive groups. By adjusting the HA concentration, cross-linker concentration, or the ratio between them, the hydrogel’s properties, such as mechanical strength and degradation rate, can be controlled. These parameters influence the density of cross-linking points within the hydrogel network, ultimately impacting its physical characteristics [55]. Yoo et al. demonstrated the synthesis of cross-linked hydrogels by combining three components: HA-Mal, Gel-Mal, and a cross-linker known as PEGDSH (PEG-dithiol). These components were utilized to create a network comprising macromolecular polysaccharides and proteins, as visualized in Figure 3. The cross-linking process in this biomaterial relies on the Michael addition reaction, involving the thiolated groups on the PEG and the maleimide groups on the HA-Mal and Gel-Mal [27].

#### 2.1.4. Esterification

**Figure 4:**
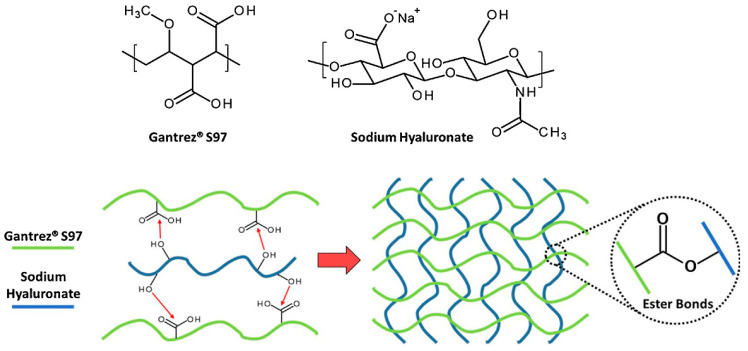
Chemical structures and proposed cross-linking mechanism of Gantrez^®^ S97 and sodium hyaluronate. Reprinted with permission from ref. [29]. 2018, Elsevier.

Esterification is a chemical reaction that involves the formation of an ester by reacting an alcohol with a carboxylic acid. In this case, esterification is used to cross-link HA molecules by forming ester bonds between them [36,56]. The process of esterification transforms HA into a hydrogel material by adding ester groups, creating cross-links that give it a gel-like texture. Bedini et al. used EDC/hydroxybenotriazole (HOBt) under heterogeneous conditions to cross-link HA networks, reducing byproducts and allowing various HA forms to be processed prior to cross-linking while retaining the initial molecular weight. This approach was expected to better exploit HA’s bioactivity for tissue regeneration compared to current methods [30]. Larrañeta et al. [29] prepared the creation of HA-based hydrogels cross-linked with poly (methyl vinyl ether alt-maleic acid) (Gantrez^®^ S97) using thermal and microwave methods (Figure 4). These hydrogels show great potential as materials for wound care, drug delivery, and medical applications. A solvent-free process produces HA hydrogels without using any solvents. In this process, solid HA powder is mixed with a cross-linking agent and other components under specific conditions like temperature, pressure, shear, etc.

#### 2.1.5. Diels–Alder (D-A) Cross-Linking

**Figure 5:**
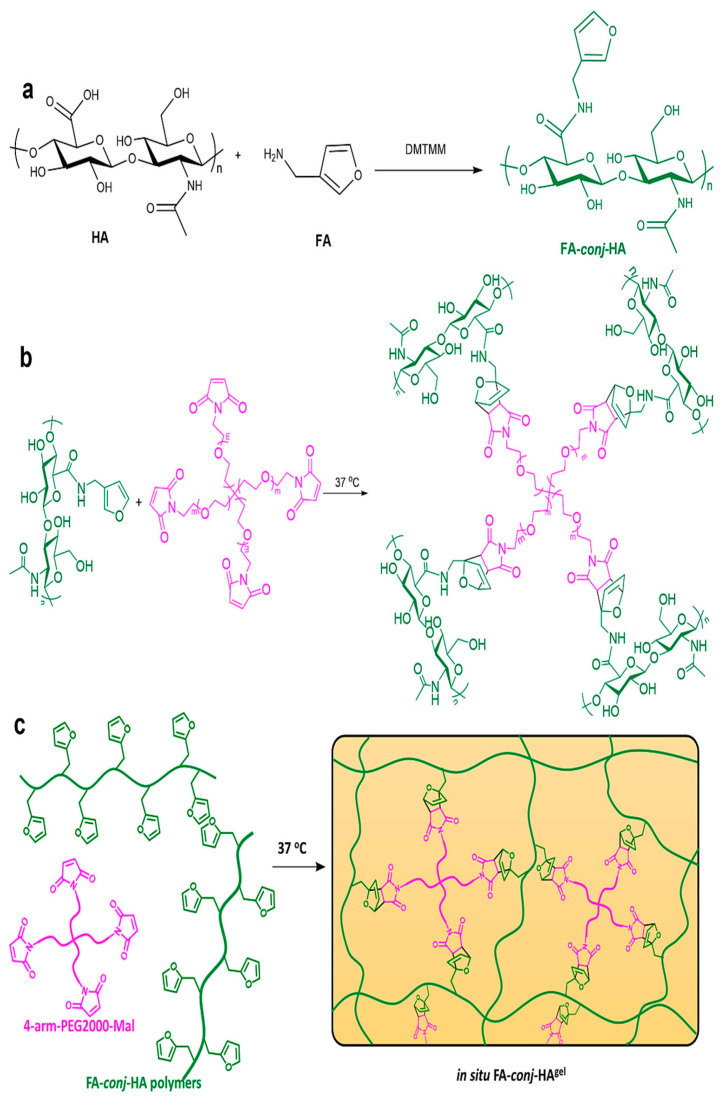
Illustrates the schematic representation of FA-conj-HA gel. The synthesis route of FA-conj-HA polymers is shown in (**a**), while the cross-linking route of FA-conj-HA gel is depicted in (**b**). In (**c**), the FA-conj-HA solution undergoes cross-linking using 4-arm-PEG2000-Mal through a D-A reaction, leading to the creation of viscoelastic hydrogels. Reprinted with permission from ref. [31]. 2024, Elsevier.

The D-A cycloaddition is a method of biomaterial conjugation used to create tools for applications [57]. It is a biocompatible one-step reaction that makes use of unique functional groups not found in natural biopolymers. It does not need catalysts and avoids generating side products, making it an ideal “click reaction” [58]. The D-A reaction involves conjugated dienes reacting with molecules containing double or triple bonds to form hexatomic cyclic compounds. Researchers have utilized aqueous D-A chemistry to conjugate a natural polysaccharide hydrogel for charged drug delivery through electrostatic interactions [31]. In their study, FA-conj-HA polymers were created by linking furfuryl amine (FA) onto HA chains, as shown in Figure 5. An injectable FA-conj-HA gel was produced by cross-linking FA-conj-HA solution with 4-arm-PEG2000-Mal. After injection, the D-A reaction between FA-conj-HA polymers and 4-arm-PEG2000-Mal was sped up at body temperature, leading to the formation of non-flowing viscoelastic hydrogels at the administration sites. The reactivity of the furan diene in the D-A reaction depends on its electron density. Furans with higher electron density, being more electron-rich, will be more reactive as dienes. Conventional furans contain an oxygen atom within the five-membered ring, which is electron-donating via its lone pair electrons, thereby enhancing the reactivity of the diene. This is in contrast to the case in which dienes that contain electron-withdrawing groups would not be favorable for reaction with electron-poor dienophiles. This results in furans typically displaying only moderate reactivity. However, adding an electron-donating methyl group increases the furan’s electron density. The methyl group donates electrons to the furan ring via an inductive effect, upregulating the electron density at the diene site. A furan with elevated electron density functions more effectively as a diene, undergoing the D-A reaction more readily with various dienophiles [59,60]. This translates to augmented reactivity and improved gelation properties when polymerizing methyl-substituted furans compared to unmodified furans. The precise placement and number of methyl groups allow for the tuning of the D-A reaction kinetics and products formed by rendering the furan more or less reactive as needed [61]. Recently, click chemistry has been seen as a promising approach to designing covalently cross-linked hydrogels [62]. Specifically, the inverse electron demand Diels–Alder (IEDDA) reaction between Nb functional groups and Tz functional groups is seen as a highly biocompatible method for creating hydrogels. Researchers have showcased the creation of photodegradable hydrogels and the mechanism of drug release [32,63]. The IEDDA click reaction between HA-Nb and DCOUM-PEG-DTz cross-linker yielded injectable hydrogels [64]. The coumarin linkages could be broken down after NIR irradiation, undoing the cross-linking or degrading the hydrogels, and releasing the encapsulated DOX.

#### 2.1.6. Photo Cross-Linking

**Figure 6:**
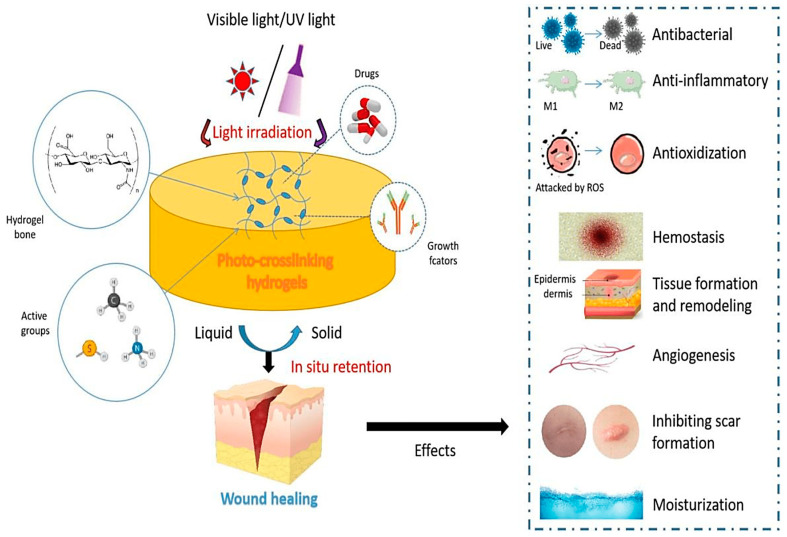
The schematic illustrates the design and the impact of photo-cross-linking hydrogels on wound healing. Reprinted with permission from ref. [66]. 2022, MDPI.

Photo cross-linking HA hydrogel is a technique that utilizes photochemical reactions to create a three-dimensional network of HA chains [65]. By introducing a photo-initiator into an HA solution, this process harnesses light-activated chemical reactions to form cross-links among HA molecules, resulting in the formation of a hydrogel [33]. Specifically, the photo-initiator absorbs light, typically ultraviolet (UV) or visible light, and undergoes activation, generating reactive species. These reactive species then engage in reactions with functional groups on HA chains, establishing new covalent bonds and thereby constructing a robust, intricate cross-linked framework within the hydrogel [34,66]. The resulting HA hydrogel boasts high water content and biocompatibility, rendering it suitable for an array of biomedical [66,67] and tissue engineering [68] applications. Significantly, the hydrogel matrix can offer structural support for cells, emulate the characteristics of the ECM, and facilitate the controlled release of bioactive molecules. Ma et al. discuss the effects of photo-cross-linked hydrogels on promoting wound healing and provide details about the animal model used for verification, as depicted in Figure 6 [66].

#### 2.1.7. Miscellaneous Cross-Linking Methods

##### Thiol-ene Click

Thiol-ene “click” chemistry has been extensively studied and proven to be a potent method for creating innovative materials in polymer chemistry and nanotechnology as well as for crafting multifunctional surfaces in a modular manner. Many drawbacks have been tackled using thiol-ene click chemistry under gentle conditions with high efficiency. The benefits of using this method include resistance to oxygen, no need for initiators (e.g., photo-initiators or other free-radical initiators), operation in aqueous solutions (e.g., water and buffers) under physiological conditions, and the creation of harmless byproducts that are safe for cells. Specifically, a type of Michael-type thiol-ene click chemistry that relies on (meth)acrylate and thiol functional groups introduced into the polymer backbone, known as “thiol-(meth)acrylate” addition, is highlighted. It can be utilized as a mild and biocompatible reaction to fabricate “clickable” hydrogels in situ, which are favorable for cell culture and beneficial for biomedical applications [36,69]. Korogiannaki et al. enhanced the quality of poly (2-hydroxyethyl methacrylate)-based contact lenses through surface modification by grafting a hydrophilic HA layer. The modification process involved the covalent bonding of thiolated HA to acrylated poly (2-hydroxyethyl methacrylate) via nucleophile-initiated Michael addition thiol-ene click chemistry. This alteration decreased the contact wetting angle, dehydration rate, and non-specific sorption of lysozyme and albumin on the lenses compared to unmodified lenses. In vitro tests also demonstrated the increased viability of human corneal epithelial cells on HA-modified substrates. These developed systems may enhance the surface characteristics of contact lenses and alleviate dryness and discomfort associated with their use [70]. Soiberman et al. formulated a gel for subconjunctival injection based on Hydroxyl-terminated Polyamidoamine (G4-PAMAM) dendrimer and HA cross-linked through thiol-ene click chemistry. The resulting gel contained dendrimer conjugates with dexamethasone, leading to a significant enhancement in the distribution of the glucocorticoid and resulting in improved clinical results [71].

##### Etherification

The synthesis of ethers is quite limited to a few examples due to the harsh reaction conditions (pH 13–14) often necessary for ether formation. When in contact with high pH, almost all hydroxyl groups are deprotonated and become more nucleophilic compared to deprotonated carboxyl groups. This is why various epoxides preferably react with hydroxyl groups. Currently, 1,4-butanediol diglycidyl ether (BDDE) stands out as the most promising diepoxide for forming ether linkages due to its easy availability and ability to break down into non-cytotoxic fragments. The benefit of the reaction with divinyl sulfone (DVS) is that it occurs at room temperature, minimizing the degradation in alkaline solutions when compared to higher temperatures. One of the most commonly used reagents for creating ethers, apart from reactions with bisepoxides, is DVS [36]. DVS is fascinating due to its high reactivity, leading to the formation of ether bonds through a simple, easy, and reproducible process without the need for organic solvents. Andrade del Olmo et al. prepared injectable hydrogels of HA-DVS to study the release of various antibiotics with acetylsalicylic acid (ASA) from biocompatible injectable hydrogels, aiming to reduce bacterial infections caused by Staphylococcus aureus [72,73]. Zhang et al. devised a method to produce the HA-L-cysteine conjugate to create hydrogels in situ through native chemical ligation. The process of hydrogel formation entailed activating the -COOH group of Boc-protected L-cysteine with NHS to create the active ester. The resulting ester facilitated a reaction with cystamine dihydrochloride in an aqueous solution to form a disulfide. The reduction of the disulfide with NaBH_4_ led to a compound containing a free thiol group that was highly reactive with epoxides. The interaction with BDDE produced a compound containing an epoxide group that could react with HA hydroxyl groups via an ether linkage. This approach of obtaining HA-L-cysteine conjugates allows the retention of the free carboxyl group of HA, potentially enabling the modification of HA with bioactive molecules to create hydrogels suitable for tissue engineering and repair applications [25].

##### Amidation

**Figure 7:**
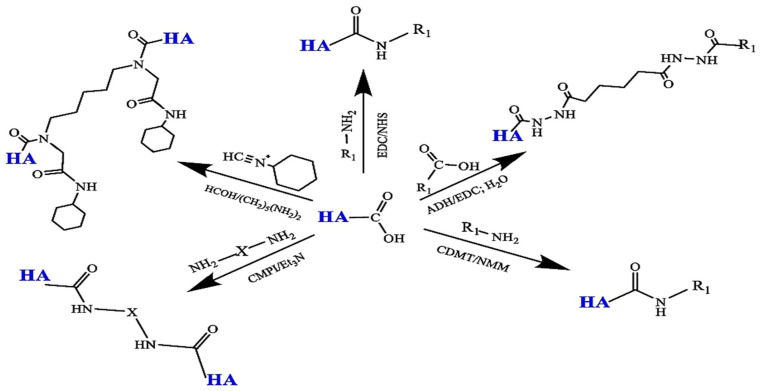
The amidation reactions of the -COOH group in chemical modifications of HA. Reprinted with permission from ref. [36]. 2024, Elsevier.

Amidation is a common reaction used to synthesize conjugates of various bioactive molecules with HA. Activators like carbodiimides, 2-chloro-1-methylpyridinium iodide (CMPI), 2-chloro-4,6-dimethoxy-1,3,5-triazine (CDMT), etc., are often employed. Micale et al. developed bioconjugates of HA and pentamidine for targeted drug delivery in treating leishmaniasis. They utilized a “triazine-activated amidation” method with CDMT and 4-methylmorpholine (NMM) as activators for binding to amino groups, testing the conjugates against the parasite [35]. Another technique involves amidation using CMPI as an activator described by Magnani et al. The reaction takes place in anhydrous dimethylformamide to minimize hydrolysis. The HA sodium salt is converted to TBA for solubilization, reacting with CMPI to form an amide bond and a cross-linking agent. Despite the need for an organic solvent and additional steps, this method is efficient due to requiring a small number of reagents [74]. Conversely, amidation with EDC is pH-sensitive. It activates the carboxyl group in a mild acidic environment and works best at a high pH. However, the hydrolysis of EDC at high pH can hinder the reaction, especially when conjugating high-pKa amines. The advantage of the EDC method is its ability to be carried out in water without pretreatment, maintaining the molecular weight and avoiding polymer chain cleavage [36]. The options for the modification of the carboxyl group HA’s amidation are displayed in Figure 7.

##### Hydrazone Linkage

**Figure 8:**
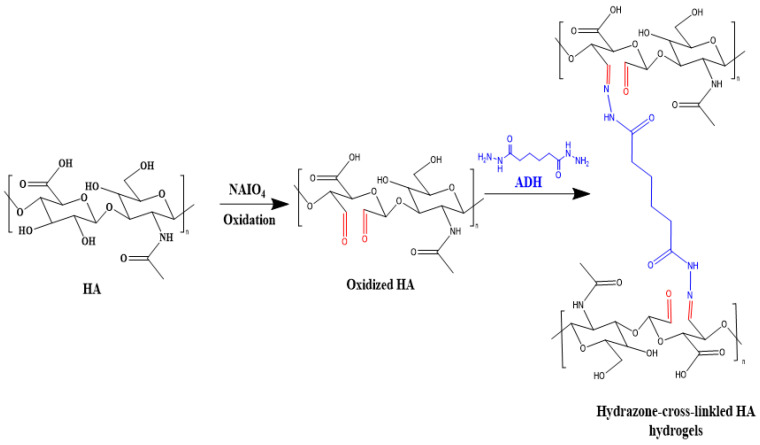
Oxidation of HA and hydrogel formation via hydrazone cross-linking.

A hydrazone linkage in HA, also known as HA hydrazide, is a type of chemical modification used to add specific functionalities or create conjugates for different biomedical applications. Hydrazone linkages are created via the reaction between a hydrazide group (-NHNH_2_) and an aldehyde or ketone group, resulting in a stable covalent bond (Figure 8) [75,76]. The original hydrazide modification used adipic acid dihydrazide (ADH) initially, followed by other mono- and polyhydrazides, to develop living HA derivatives. HA-ADH is commonly used as it can form hydrazone linkages with ketones and aldehydes, as well as acylhydrazides with acylating agents, allowing for cross-linking, the incorporation of hydrophobic groups, the attachment of drugs or polypeptides, and shows great potential in biomedical applications due to its non-cytotoxic nature [37]. To prepare HA for hydrazone cross-linking, it can be modified with either aldehyde groups or ADH functional groups (HA-ADH). To introduce aldehyde groups to HA, the polymer undergoes oxidation in the presence of sodium periodate (NaIO_4_). Alternatively, to attach ADH to HA, HA is mixed with ADH in the presence of EDC at a pH of 4.75. Zhang et al. utilized quaternized carboxymethyl chitosan, aldehyde hyaluronic acid, ADH, and anhydrous calcium chloride as raw materials to produce injectable multifunctional hydrogels with remarkable mechanical properties, self-healing abilities, pH responsiveness, antibacterial properties, and a high drug loading capacity due to the synergistic effects of their imine bonds, acylhydrazone bonds, and coordination bonds [39]. 

#### 2.1.8. Identification and Quantification of Functionalized HA

##### Fourier-Transform Infrared Spectroscopy (FTIR)

FTIR is a technique employed for the analysis of the molecular structure and composition of substances, including HA hydrogels [77]. In the context of HA hydrogels, FTIR plays a crucial role in providing insightful information about chemical bonds, functional groups, and overall molecular characteristics [78]. By exposing the HA hydrogel sample to infrared radiation, FTIR measures the absorption and transmission of radiation at varying wavelengths. This enables the identification of distinct molecular vibrations and bonds within the hydrogel [79,80]. Researchers can compare the FTIR spectra of HA hydrogels with reference spectra or control samples to evaluate the impact of different preparation methods, modifications, or environmental conditions on the hydrogel’s molecular structure and properties.

##### Proton Nuclear Magnetic Resonance (^1^H NMR)

**Figure 9:**
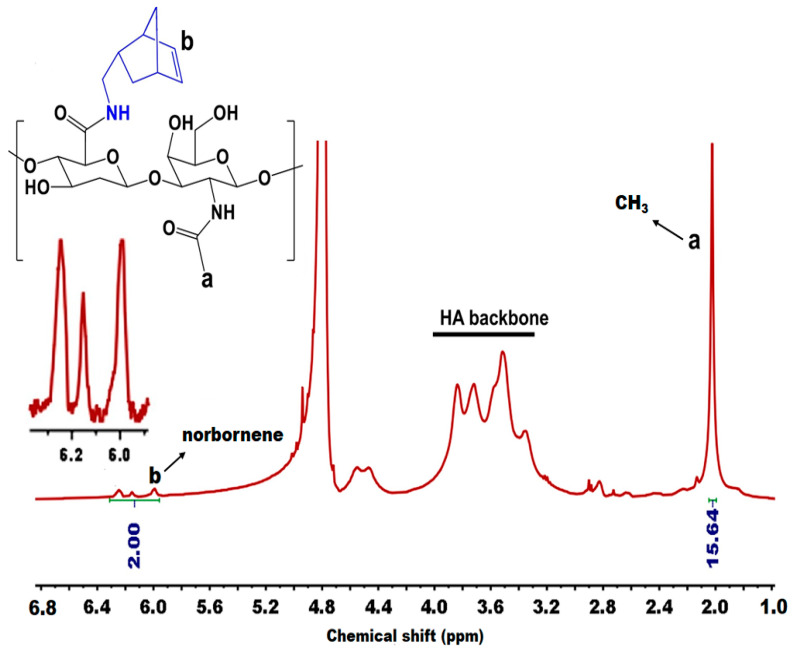
^1^H NMR characterization of the precursor of HA-Nb in D_2_O. Reprinted with permission from ref. [83]. 2022, Elsevier.

NMR is an invaluable tool for elucidating the structure and makeup of polymers, encompassing the identification of functional groups, monomer sequences, stereochemistry, conformations, and bonding configurations. This aids in the thorough characterization of synthesized polymers [64,81]. ^1^H NMR plays a pivotal role in the detection and identification of residual monomers, solvents, catalysts, or other impurities within synthesized polymers. The in situ ^1^H NMR monitoring of reactions facilitates step-by-step observation, offering valuable insights into reaction mechanisms and intermediates during polymer synthesis [63,82]. Jo et al. [83] functionalized HA with 5-norbornene-2-methylamine by employing a carbodiimide coupling reaction using EDC and NHS. The resulting HA-Nb precursor is primed for an IEDDA click reaction. The structure of the synthesized HA-Nb was probed using ^1^H NMR. The degree of substitution (DS) of Nb was assessed by integrating the protons of the Nb moiety and the methyl protons of the HA moiety. By analyzing the ^1^H NMR spectrum (Figure 9), the DS of Nb was determined to be 20% based on the integration of the Nb protons from 5.9 to 6.3 ppm and the methyl protons of HA at 2.0 ppm. 

##### Carbon-13 Nuclear Magnetic Resonance (^13^C NMR)

**Figure 10:**
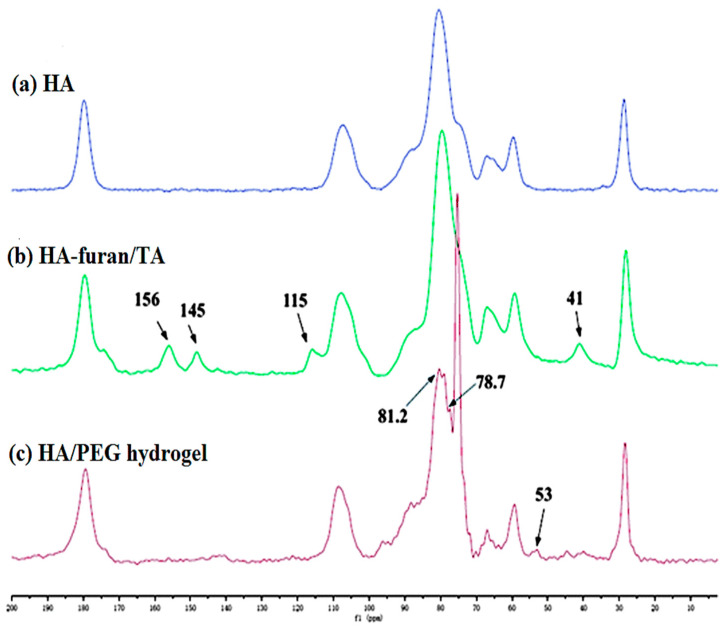
^13^C-NMR spectra (400 MHz) of (**a**,**b**) HA–furan/TA and (**c**) HA/PEG hydrogel are solid, indicating the presence of both D-A click chemistry and enzymatic cross-linking processes. Reprinted with permission from ref. [86]. 2014, RSC.

^13^C NMR is a powerful analytical technique used to investigate the carbon environment in molecules, including complex polymers like HA. The ^13^C NMR spectrum of HA provides a wealth of information about the structure and composition of this crucial biomolecule. The anomeric carbon signals confirm the presence and identity of the repeating disaccharide units, made up of N-acetyl-D-glucosamine and D-glucuronic acid. By examining the positions and chemical shifts of these anomeric signals, the glycosidic linkages between the monosaccharide units can be understood [84,85]. Additionally, the distinct signals corresponding to the acetyl carbon and the carboxyl carbon enable the identification of the key functional groups, the N-acetyl and carboxyl groups which are fundamental to the structure and properties of HA. Moreover, the ^13^C NMR spectrum offers insights into the chemical environment and substitution patterns of the monosaccharide rings, providing a comprehensive understanding of the structural features of the complex polysaccharide [84]. Yu et al. modified HA by introducing furan and tyramine (TA) functional groups. The HA/PEG hydrogel was created through enzymatic cross-linking and sequential D-A click chemistry [86] (Figure 10).

##### Titration

The titration of HA involves a careful addition of a titrant, such as an acid or base solution, to analyze its acid–base properties by tracking pH changes. HA, a polyelectrolyte, has multiple ionizable carboxyl groups along its polymer structure. These groups can either accept or donate protons, giving HA both acidic and basic characteristics [87,88]. Through titration, we can identify the pKa values of these carboxyl groups, shedding light on HA’s ionization behavior and pH-dependent charge. The procedure entails adding precise amounts of an acid or base solution to the HA solution and monitoring pH changes using a pH meter. The resulting titration curve, which shows pH against titrant volume, reveals information about HA’s buffer capacity, charge density, and pH-responsive properties. Variations in the titration curve for HA may stem from factors like molecular weight, cross-linking level, and environmental factors. By analyzing the curve and determining the endpoint titrant volume, the original HA concentration in the solution can be calculated [89].

### 2.2. Physical Cross-Linking

The physical cross-linking of HA hydrogel involves the creation of a 3D network in the hydrogel matrix using non-covalent interactions. Unlike chemical cross-linking, which relies on covalent bonds, physical cross-linking utilizes reversible interactions like hydrogen bonding, electrostatic interactions, and polymer chain entanglements. These interactions give the hydrogel matrix its structural integrity and determine mechanical properties like stiffness and elasticity [90]. Physical cross-linking enables the production of hydrogels with adjustable characteristics, making them suitable for diverse biomedical applications such as tissue engineering, drug delivery, and wound healing. This approach also offers the potential for a more biocompatible environment [91].

#### 2.2.1. Temperature-Induced Gelation

**Figure 11:**
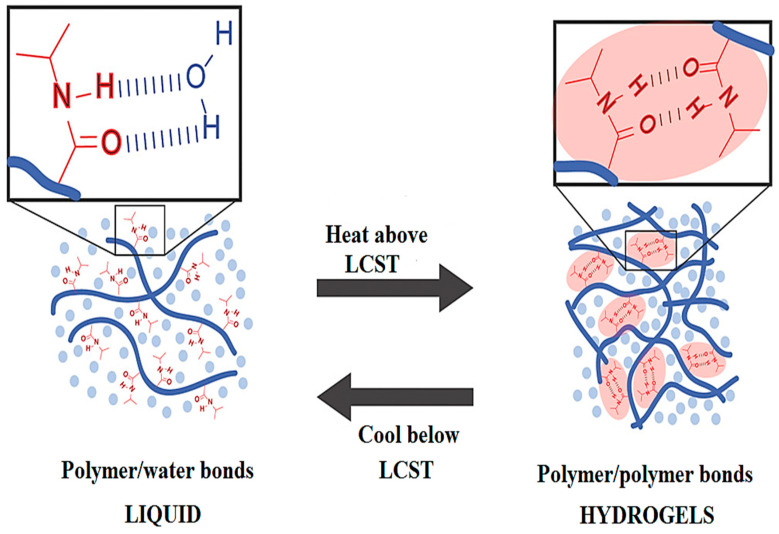
The schematic of HA-PNIPAAm demonstrates the amide groups of PNIPAAm forming hydrogen bonds with water below the LCST (lower critical solution temperature) and forming hydrogen bonds with each other above the LCST, thereby the formation of hydrophobic microdomains and the transformation of the material into a physically cross-linked hydrogel. Reprinted with permission from ref. [41]. 2018, Wiley.

Temperature-induced gelation is a fascinating phenomenon observed in hydrogels, particularly HA hydrogel, wherein a transition occurs from a liquid to a gel state in response to changes in temperature [77]. During this process, the polymer chains of HA are altered or cross-linked, leading to the formation of a three-dimensional network structure. Unlike chemical reactions, physical interactions, such as hydrogen bonding and hydrophobic interactions, play a crucial role in achieving this cross-linking. Initially, at lower temperatures, the HA solution remains in a liquid state as the polymer chains lack sufficient entanglement or association. However, as the temperature rises, these chains gradually interact and entangle with each other, resulting in the gelation process and the creation of a gel network [92]. Remarkably, this process is reversible, allowing the gel to transform back into a liquid state when the temperature drops below the critical gelation temperature. In their study, Ekerdt et al. introduced an adaptable biomaterial with thermos-responsive properties, which comprises HA and PNIPAAm (refer to Figure 11). The key characteristic of this biomaterial is its lower critical solution temperature (LCST), representing the temperature at which the polymer blend becomes insoluble in water. When the temperature surpasses the LCST, the PNIPAAm polymer blocks undergo microphase separation, leading to the formation of a hydrogel [40,41]. This property proves advantageous for applications that necessitate injectability or the ability to reshape the gel material. HA hydrogels formed via temperature-induced gelation exhibit a range of desirable characteristics, including high water content, biocompatibility, and ease of manipulation [93]. On the other hand, methylcellulose, derived from plant sources, is a modified cellulose polymer that exhibits thermo-reversible or “inverse-thermal” gelation properties. Specifically, it liquefies at lower temperatures and gels at higher temperatures near body temperature. By combining methylcellulose with HA in optimal ratios, their individual characteristics combine to produce a co-polymer blend that also demonstrates inverse-thermal gelation. At low temperatures, the HA/methylcellulose mixture remains freely flowable, allowing it to be conveniently injected or administered surgically. However, as its temperature rises towards 37 °C or body heat, it starts to physically cross-link and forms a gel scaffold [94,95]. This temperature-induced transition permits the co-polymer blend to be delivered minimally invasively in liquid form, after which it sets into a three-dimensional network within the body, which is well suited for tissue regeneration applications [96]. Investigations into tissue regeneration [97], wound healing [96], and drug delivery [77] have utilized this inverse-thermal HA/methylcellulose system, owing to its gelation behavior and HA content producing a supportive scaffold conducive to tissue growth.

#### 2.2.2. Covalent Augmentation

**Figure 12:**
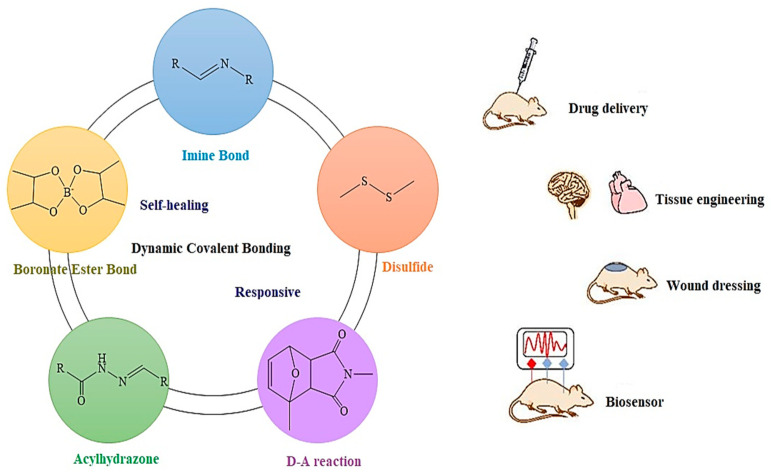
An Overview of Dynamic Covalent Bonding and their biomedical application. Reprinted with permission from ref. [105]. 2020, Elsevier.

Covalent augmentation refers to a method of enhancing the physical properties of HA through chemical modifications or cross-linking [98]. This process involves introducing chemical changes or cross-linking agents to the HA hydrogel. By incorporating functional groups or reactive molecules into the HA matrix, they can interact and form covalent bonds with each other [99]. These connections between the HA chains improve the mechanical characteristics of the hydrogel, such as its strength, elasticity, and stability [100]. For instance, by using cross-linking agents like polyethylene glycol diacrylate (PEGDA) in the presence of a radical initiator, the mechanical properties of the hydrogel can be enhanced [42,101]. Another related study by Loebel et al. [102] explored creating a self-healing hydrogel for the controlled release of therapeutic agents by modifying HA with β-cyclodextrin and adamantine. The prepared hydrogel acted like a polymer melt when passed through a syringe, demonstrating shear thinning properties where viscosity was reduced. Additionally, after removing the shear forces, the hydrogel was able to reassemble and self-heal, with viscosity returning to its original value. To enable secondary cross-linking, HA-based polymers were further modified with methacrylate groups. This modified hydrogel showed the ability to flow when mechanical forces were applied but rapidly self-heal and regain viscosity once forces were removed, making it promising for applications requiring the controlled release of proteins and growth factors [103]. This approach allows researchers and engineers to customize the physical attributes of the HA hydrogel for specific applications. For example, covalent augmentation can elevate the load-bearing capacity of HA hydrogel for tissue engineering scaffolds [104] or enhance its viscosity for controlled drug delivery systems. Meanwhile, the modified hydrogel retains its inherent biological properties while offering improved mechanical features. Overall, the covalent augmentation of HA hydrogels holds great promise in optimizing their physical properties and expanding their potential applications in various fields of biomedicine and biomaterials [99]. Ye et al. conducted a comprehensive review of hydrogels that utilize dynamic covalent bonding, with a focus on their promising potential for biomedical applications, which is shown in Figure 12 [105].

#### 2.2.3. Freeze–Thawing

Freeze–thawing is a physical technique employed for modifying HA hydrogels. The procedure entails placing the HA solution or hydrogel in a freezer, lowering the temperature below freezing point to solidify it [43,106]. Subsequently, the solution or hydrogel is thawed by raising the temperature, usually to room temperature or higher, facilitating the melting process [107]. This freeze–thaw cycle is repeated multiple times, involving the successive freezing and thawing of the hydrogel [43,107]. As a result of this freeze–thaw process, the HA/PVA hydrogels undergo physical transformations, leading to the creation of interconnected networks and modifying their properties [43,44]. These alterations can impact the structure, porosity, mechanical strength, and release characteristics of the hydrogel. Gelation during the freeze–thaw process occurs via phase separation as the solution transitions between liquid and solid states. As water crystallizes into ice crystals during freezing, solutes such as polymer molecules are expelled from the ice phase and become concentrated in the remaining liquid fraction. This increase in polymer concentration leads to stronger polymer–polymer interactions and, in some cases, cross-linking reactions. During thawing as the ice melts, the polymer solution gels into a hydrogel network held together with physical or chemical bonds [108,109]. Cryogels are hydrogels specifically formed using this freeze–thaw or cryogelation technique. In the cryogelation process, a precursor polymer solution is frozen and then thawed to produce a gel with an interconnected porous structure. When chemical cross-linking is employed, a cross-linking agent is added to the polymer solution before freezing. During freezing, the polymers and cross-linker become concentrated in the unfrozen liquid portion as water crystallizes out. Upon thawing, the cross-linker chemically reacts with the polymer chains, covalently linking them together into a solidified, porous gel network. Cryogels synthesized via chemical cross-linking possess a high surface area and easily tunable porosity characteristics. These properties make cryogels well suited for applications such as tissue engineering, drug delivery, wound dressings, and other biomedical uses [43,110].

### 2.3. Enzymatic Cross-Linking

The enzymatic cross-linking of HA hydrogels involves the utilization of enzymes to establish chemical bonds within the HA molecules, resulting in the creation of a stable hydrogel structure [111,112]. This process significantly enhances the mechanical properties and stability of the hydrogel, making it highly applicable in various biomedical fields. Enzymatic cross-linking offers several advantages compared to traditional methods like chemical or physical cross-linking [113,114]. One significant advantage is its biocompatibility since enzymes are typically non-toxic and naturally present in the body. Multiple methods exist for the enzymatic cross-linking of HA hydrogels, with horseradish peroxidase (HRP) [45] and tyramine [46] being commonly used enzymes for this purpose. These enzymatically cross-linked HA hydrogels find suitability in tissue engineering [86,115], drug delivery systems [116], and wound healing [117] applications.

## 3. Techniques Used to Investigate the Properties of HA Hydrogels

Characterization techniques play a crucial role in comprehending the properties of HA hydrogels, which are networks of cross-linked HA molecules [118,119]. The following techniques are commonly employed to investigate these hydrogels:

### 3.1. Rheological Analysis

**Figure 13:**
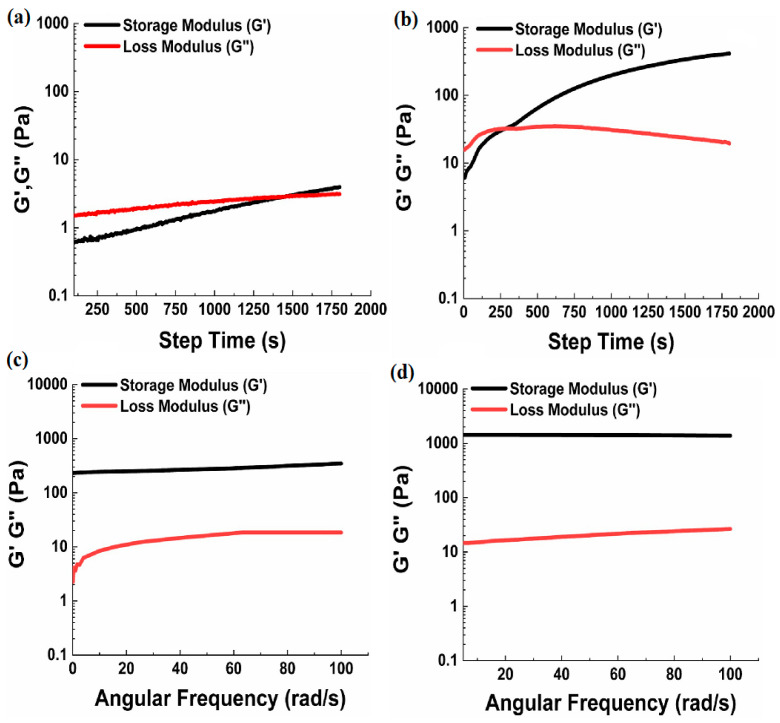
The moduli of HA/Coumarin-25 and HA/Coumarin-100 hydrogels were measured in two ways. Firstly, their moduli were measured as a function of step time (**a**,**b**), and secondly, their moduli were measured as a function of angular frequency (**c**,**d**). Reprinted with permission from ref. [64]. 2023, Elsevier.

The rheological analysis of HA hydrogels involves the examination of their mechanical and flow characteristics. Rheology, a branch of physics, focuses on the deformation and flow of materials [120,121]. Conducting rheological analysis aids in understanding the viscoelastic behavior of HA hydrogels. It helps assess stability, integrity, and response to external forces [122]. Gulfam et al. reported that a chemically cross-linked hydrogel can be created using a click reaction. The time it takes for hydrogels to undergo sol–gel transformation can be accurately determined by analyzing their storage modulus (Gʹ) and loss modulus (G″) as a function of step time using a rheometer. Varying the mole ratio of the cross-linker was found to alter the gelation times of the hydrogels. As shown in Figure 13, the hydrogel HA/Coumarin-25 (with 25% cross-linking and a 10:2.5 Nb/Tz ratio) took about 20.12 ± 5.78 min to gel, as determined via the intersection of G′ and G″ in Figure 13a. Conversely, the gelation times for hydrogel HA/Coumarin-100 (Figure 13b) decreased to 5.22 ± 0.64 min due to their higher degree of cross-linking (100%). These observations indicate that the hydrogels are injectable, providing ample time for the mixing and subsequent injection of the pre-gel solution into the body followed by solid gel formation. Furthermore, when analyzed for extended periods of time at a constant strain of 1% and angular frequency of 10 rad/s, the hydrogels showed a consistent trend in their G′ values, with a higher concentration of cross-linker leading to increased mechanical strength. Figure 13c,d displays the G′ variations of hydrogels with oscillation frequency. It is observed that increasing the feed ratios of the cross-linker resulted in an increase in the G′ of hydrogels. In Figure 13c,d, the G′ values at the initial frequency were noted as 346 and 1380 Pa for HA/Coumarin-25 and HA/Coumarin-100, respectively [64].

### 3.2. Swelling Behavior

**Figure 14:**
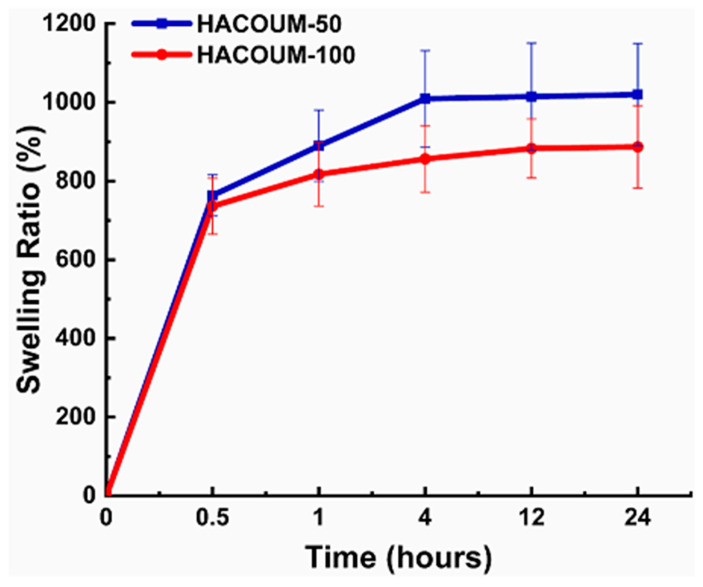
The coumarin-functionalized HA hydrogels with different molar ratios (50:100, 100:100 with respect to Nb/Tz). Reprinted with permission from ref. [64]. 2023, Elsevier.

Swelling studies are conducted to evaluate the water absorption and retention capabilities of HA hydrogels [123]. When HA hydrogels come into contact with an aqueous environment, they absorb water and undergo expansion or swelling [124]. Multiple factors influence the swelling behavior, such as the molecular weight and concentration of HA, cross-linking density [125], pH [126], temperature [127], and the presence of ions. The behavior of HA hydrogels is also affected by pH and temperature. pH variations can impact the ionization state of HA, affecting its hydrophilicity and swelling capacity. Changes in temperature can influence the kinetic energy of water molecules, affecting their movement within the hydrogel network and consequently influencing the swelling behavior [128]. Cross-linking density significantly contributes to the swelling behavior of HA hydrogels. A higher cross-linking density restricts the motion of polymer chains, resulting in reduced swelling capacity. On the other hand, a lower cross-linking density enables increased water absorption, leading to enhanced swelling [129]. The swelling ratios of hydrogels are shown in Figure 14. Both of the tested hydrogels swelled dynamically during the initial first hour. This initial rapid swelling could be due to the porous or sponge-like structures of the hydrogels, which facilitated the diffusing of water molecules into the hydrogels, as Gulfam et al. reported previously [64].

### 3.3. Morphology Examination

**Figure 15:**
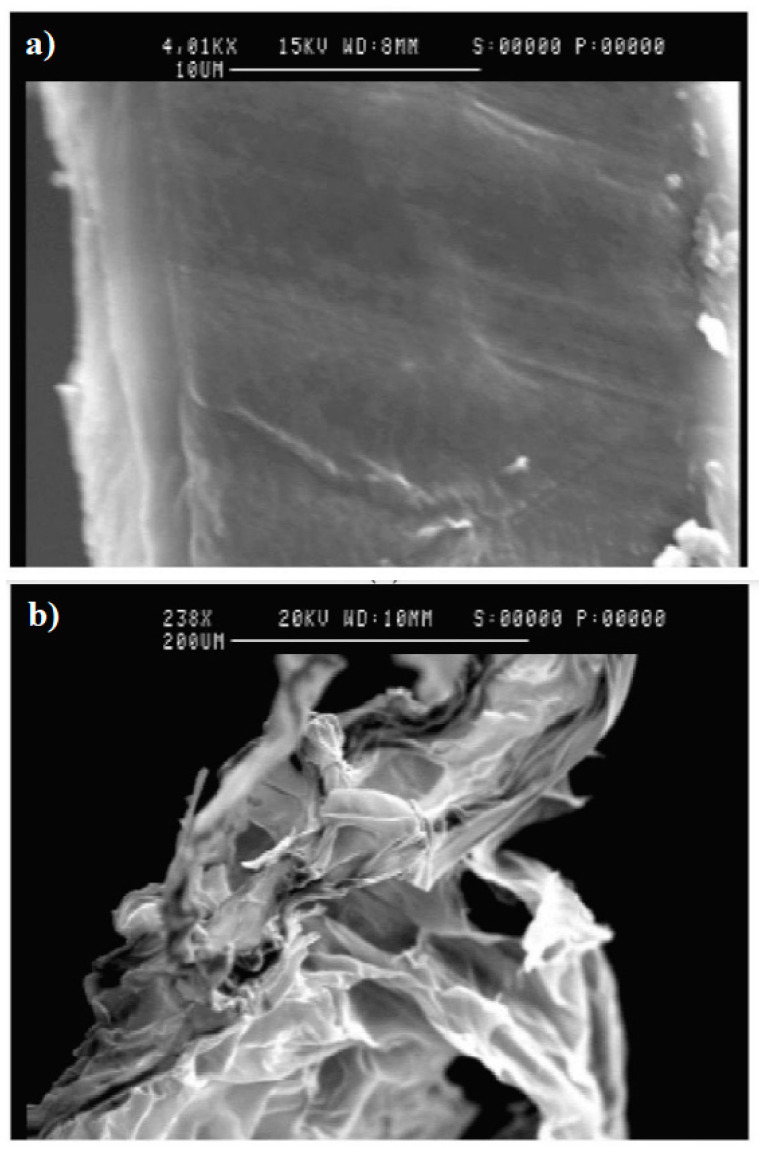
SEM images of HA hydrogel films. Cross-section image (**a**) of HA dried hydrogel film; (**b**) cross-section image of HA hydrogel film in swelling status. Reprinted with permission from ref. [132]. 2000, Elsevier.

There are several techniques available for examining the morphology of HA hydrogels. Some commonly used methods include scanning electron microscopy (SEM), transmission electron microscopy (TEM), atomic force microscopy (AFM), and X-ray diffraction (XRD). SEM [130,131] enables the capture of high-resolution images depicting the surface morphology of the hydrogel. This technique facilitates the visualization of the topography, porosity, and surface characteristics of the hydrogel structure. Luo et al. utilized SEM images to analyze the surface of HA hydrogel films. Samples of the HA hydrogel films were prepared in both dried and hydrated states, and the resulting SEM images are displayed in Figure 15. The cross-sectional image of dried HA films, shown in Figure 15a, appears flat and featureless, indicating a condensed structure when dry. On the other hand, Figure 15b displays the cross-sectional images of freeze-dried HA hydrogel films after rehydration, revealing a highly porous structure in the swollen hydrogel [132].

**Figure 16:**
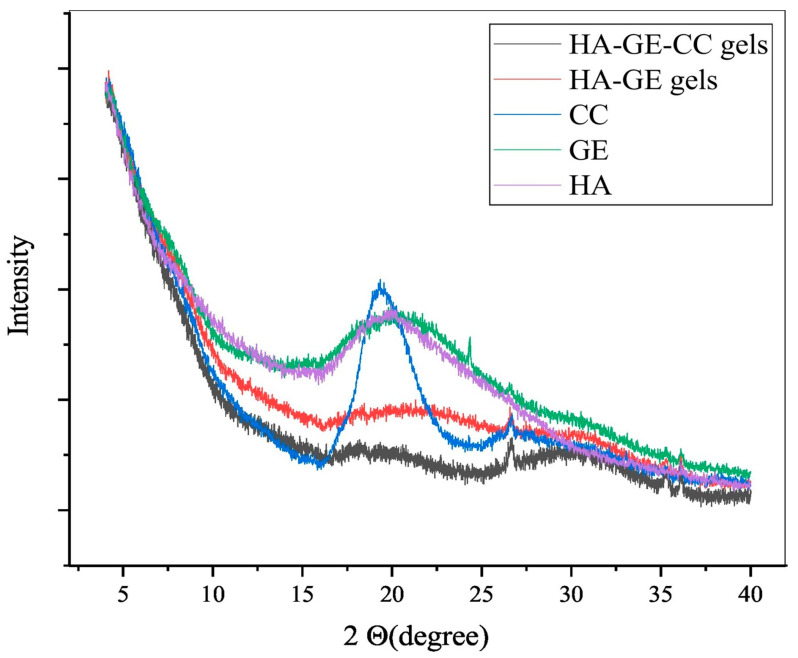
The X-ray diffraction (XRD) patterns of microspheres made of carboxymethyl chitosan and loaded with HA/gelatin hydrogels were analyzed. Reprinted with permission from ref. [79]. 2021, Elsevier.

On the other hand, TEM [133] is employed to investigate the internal structure of HA hydrogels at higher magnifications. It provides detailed information regarding the arrangement, distribution, and possible presence of internal structures or particles within the gel matrix. AFM [134], operating at the nanoscale, allows for the imaging of surface morphology and topography and offers insights into properties such as surface roughness, gel structure, and interactions with other materials. XRD [135] is capable of analyzing the crystallographic structure of HA hydrogels, providing valuable data about the composition of crystalline and amorphous phases present in the gel matrix. Yang and colleagues investigated the use of carboxymethyl chitosan microsphere-loaded HA/gelatin hydrogels for controlled drug delivery. They analyzed the XRD patterns of HA, gelatin, carboxymethyl chitosan, and hydrogels within the 2θ range of 2–40°, as depicted in Figure 16. The XRD profile of gelatin and carboxymethyl chitosan polymers showed a broad peak at 20°. The XRD scanning of gelatin revealed a wide diffraction peak near the 2θ of 21°, indicating its amorphous nature. However, the crystal structure of HA and gelatin was disrupted after the preparation of the composite gel, demonstrating the cross-linking reaction between HA and gelatin [79].

### 3.4. Thermal Analysis

**Figure 17:**
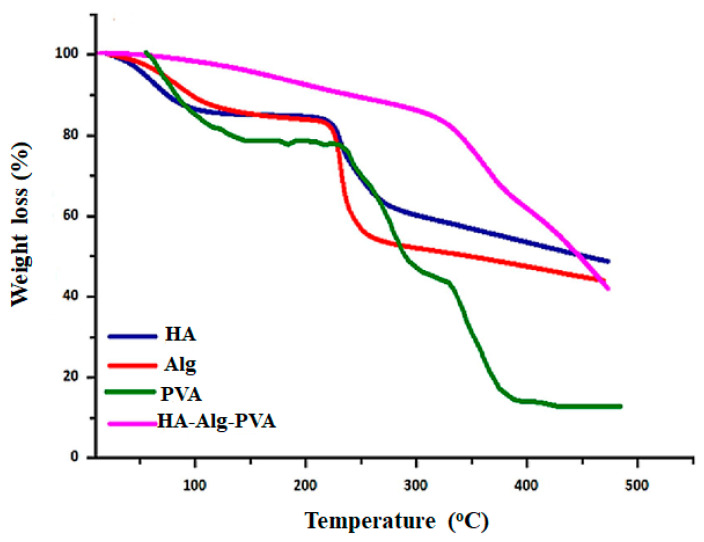
TGA analysis of HA-Alg-PVA hydrogel membrane. Reprinted with permission from ref. [139]. 2023, Elsevier.

Thermal analysis is a process used to examine the physical and chemical properties of HA hydrogels as they are exposed to temperature variations [46]. Two common techniques employed in thermal analysis are differential scanning calorimetry (DSC) and thermogravimetric analysis (TGA). DSC [136] measures the heat flow during thermal transitions within the hydrogel, providing insights into changes in heat content (Enthalpy) concerning temperature. This reveals important phase transitions like gelation, melting, or degradation. On the other hand, TGA [80,137] tracks changes in a sample’s weight as temperature changes, enabling the assessment of thermal stability and decomposition characteristics of HA hydrogels [138]. The thermogravimetric tests were conducted on the samples to assess their water content and degradation process as performed by Khaliq et al. The TGA curve of HA (Figure 17) showed a weight decrease between 75 and 100 °C, indicating water loss of 15%. At 250 °C, thermal modification began and resulted in a significant mass loss (20%) until approximately 300 °C. A third-phase weight loss of approximately 40% is observed at temperatures above 300 °C, which corresponds to residual decomposition. Both DSC and TGA analyses revealed the typical thermal behavior of polysaccharides, with an endothermic peak due to water evaporation and a peak indicating degradation [139]. These thermal analysis methods facilitate a comprehensive understanding of the thermal behavior, stability, and overall performance of HA hydrogels for research purposes.

## 4. Utilization of Hydrogels Based on HA

Due to its biocompatible nature, adjustable characteristics, biodegradability, and inherent bio-functionality, HA hydrogels have gained significant prominence in various biomedical domains. In tissue engineering applications, HA hydrogels can be utilized as scaffolds to facilitate cartilage, bone, skin, and nerve regeneration. Their tunable properties allow for the mimicking of the mechanical and biochemical cues of native ECM. HA hydrogels have immense potential and play a crucial role in materials for regenerative medicine. As drug delivery vehicles, HA hydrogels offer the protected encapsulation of labile therapeutics and the ability to control release kinetics. They have been explored to deliver anti-inflammatory drugs for arthritis treatment, antibiotics for wound healing, and chemotherapeutics for cancer. HA hydrogels also show promise in ophthalmic applications such as artificial tear substitutes to lubricate dry eyes and as cell carriers for corneal tissue regeneration following injuries. Their moisturizing ability aids wound healing by maintaining a hydrated environment. Furthermore, HA hydrogel dressings have been developed for chronic wounds like pressure ulcers and diabetic foot ulcers. The dressings provide a protective barrier while promoting the autolytic debridement of non-viable tissue and cell repopulation. Their tunable properties are conducive to personalized wound care needs. HA hydrogels also enable biofabrication through additive manufacturing techniques like the 3D bioprinting of complex tissues [117,140].

### 4.1. Tissue Engineering

**Figure 18:**
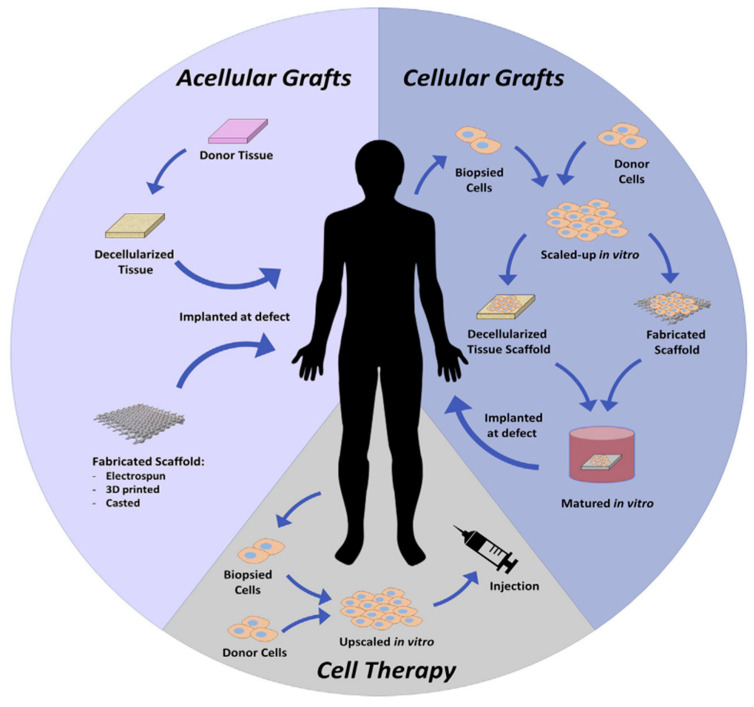
Tissue engineering strategies for regeneration can involve different approaches. In acellular methods, recipient-derived or artificial biomaterial structures without any cells are placed into the patient’s body to enhance natural regeneration processes. Cellular techniques utilize patient-specific or donor cells to populate and develop a framework before implantation. Cell therapy, on the other hand, involves administering intended cell types and biological populations directly to the patient without the use of scaffolds. Reprinted with permission from ref. [141]. 2020, Elsevier.

Traditional tissue engineering involves two main approaches: (1) transplanting in vitro-grown tissue made up of an artificial matrix with cells and growth factors and (2) regenerating tissue in situ using an artificial matrix and growth factors as a template to stimulate host cell regeneration within the living organism [12,139,141] (see Figure 18). Among the various biomaterials employed in tissue engineering, HA-based hydrogels have garnered considerable attention, owing to their exceptional properties and versatile applications [142]. HA-based hydrogels offer several advantages in the context of tissue engineering. Primarily, they create a supportive microenvironment conducive to cell adhesion, proliferation, and differentiation [143,144]. HA molecules also interact with cell surface receptors, influencing cellular behavior such as migration and signaling pathways. Moreover, HA hydrogels can be customized to exhibit distinct physical and biochemical attributes, such as stiffness, porosity, and degradation rate, to accommodate specific tissue requirements [145]. They can be combined with bioactive molecules, growth factors, and cells to augment tissue regeneration potential. In the field of tissue engineering, HA-based hydrogels have been extensively explored across diverse applications, including cartilage [146], skin [139], bone [147], and nerve [148] tissue engineering. These hydrogels have showcased promising outcomes in fostering cell viability, tissue formation, and seamless integration with the host tissue. Nevertheless, challenges persist with HA-based hydrogels, such as achieving optimal mechanical properties, controlling degradation rates, and ensuring long-term stability. Over the past decade, advancements in tissue engineering have introduced innovative cell sources, engineering materials, and tissue architecture techniques. These developments have resulted in the creation of engineered tissues that more effectively restore, maintain, enhance, or substitute biological tissues. Current research endeavors are focused on refining the design and functionality of HA-based hydrogels to enhance their performance and broaden their range of applications in tissue engineering and regenerative medicine [149,150]. Also, HyStem is a hydrogel containing HA as a key ingredient. It was one of the first commercially available HA hydrogels used in tissue engineering [151]. HyStem was introduced utilizing thiol-modified HA for cross-linking to create hydrogels. The innovative thiol-HA chemistry of HyStem allows it to be injected or applied locally, transforming into a gel at the site. Its cross-linking capabilities enable HyStem to be delivered as a liquid before gelling in place, offering minimally invasive treatment to target tissues. This sets it apart from previous HA hydrogels that required preformation [152,153,154].

### 4.2. Drug Delivery Systems

**Figure 19:**
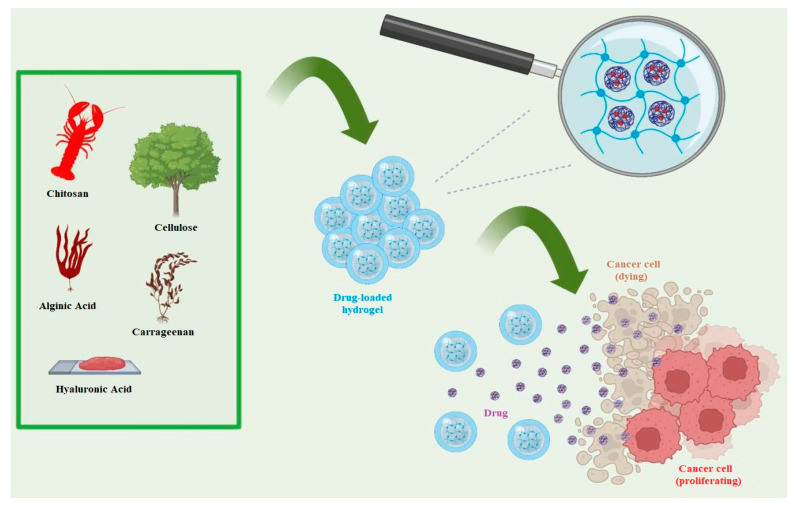
Polysaccharide-Based Hydrogels and Their Application as Drug Delivery Systems in Cancer Treatment. Reprinted with permission from ref. [159]. 2023, MDPI.

HA hydrogels have attracted considerable interest and focus in the field of drug delivery systems due to their exceptional characteristics and wide range of applications [155]. They play diverse roles in drug delivery systems, including enhancing drug stability [139], enabling controlled drug release [138], and facilitating targeted delivery [64]. HA, a naturally occurring biomolecule in the body, contributes to the high biocompatibility and low immunogenicity of the derived hydrogels. This biocompatibility minimizes the risk of adverse reactions and ensures safe and well-tolerated drug delivery [142]. The incorporation of drugs into the hydrogel matrix provides protection against degradation, enzymatic activity, and rapid clearance, resulting in improved drug stability and sustained release. Tailoring the porosity and swelling properties of the hydrogel allows for precise control over the release kinetics, enabling gradual drug distribution over an extended period [156,157]. Moreover, HA hydrogels can be functionalized with specific targeting ligands or antibodies to achieve targeted drug delivery. By attaching these elements to the hydrogel structure, the drug-loaded hydrogel particles selectively bind to receptors on target cells or tissues, concentrating the drug at the desired site and reducing off-target effects [158]. This targeted approach enhances drug efficacy and minimizes potential side effects. In summary, HA hydrogels possess unique properties that make them highly suitable for drug delivery systems. Their versatility and ability to improve drug stability, control release, and enable targeted delivery make them a promising avenue in pharmaceutical development. Parisi and colleagues outlined the proper procedure for loading and administering the medication shown in the illustration [159] (Figure 19).

### 4.3. Wound Healing

**Figure 20:**
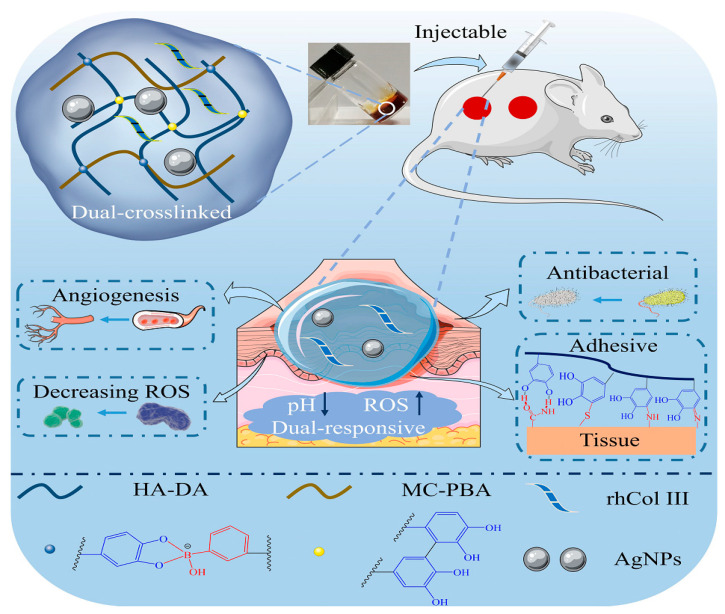
Illustration outlining the process and roles of injectable multifunctional hydrogel. Reprinted with permission from ref. [164]. 2022, Elsevier.

Wound healing typically involves the coagulation, inflammation, proliferation, and remodeling phases, representing a complex and gradual process. Factors like scalding burns, mechanical damage, diabetes, vascular diseases, and malignant tumors can easily lead to skin trauma [6]. Hydrogel stands out as the most popular material for wound dressings due to its exceptional properties, offering substantial research potential in the development of new dressing types. However, traditional single-component hydrogel dressings often lack adequate mechanical strength, hydrophilicity, and versatility, which restrict their utility in wound care [160]. To address this, integrating modified or composite hydrogel matrices with bioactive substances, medications, and nanomaterials through thoughtful design and preparation can yield innovative multifunctional hydrogel dressings with superior characteristics to enhance wound healing [161]. During the early stages of healing, the presence of HA facilitates keratinocyte migration and proliferation, aiding in nutrient transport and waste removal. HA plays a vital role in maintaining proper wound hydration through its affinity to water [162]. Studies have demonstrated that HA can expedite the healing process of skin wounds in various animal models and even in challenging chronic wounds like diabetic foot ulcers in humans. Wang et al. [163] successfully synthesized HA-Dopamine@recombinant human collagen type-III (rhCol) hydrogel, leveraging the catechol group oxidation via an H_2_O_2_/HRP catalytic system. This unique hydrogel, combining the benefits of HA-Dopamine (HA-DA) and rhCol, exhibited exceptional properties. In Figure 20, Long et al. [164] demonstrate a remarkable injectable hydrogel endowed with self-healing properties, antibacterial and antioxidant activities, and tissue adhesion. This achievement was realized through the utilization of HA-DA and methylcellulose grafted with phenylboric acid.

### 4.4. Ophthalmology

HA hydrogels have garnered significant interest in the realm of ophthalmology, owing to their exceptional properties that find extensive application in medical contexts (Table 2) [48]. These hydrogels have been extensively investigated for their use in corneal tissue engineering and regeneration. By providing a supportive structure, the hydrogel scaffold facilitates the growth of corneal cells, thereby aiding in corneal wound healing and tissue restoration [47,165]. Moreover, HA hydrogels exhibit great promise as drug delivery vehicles for ophthalmic medications. With the ability to encapsulate drugs, they can be applied to the ocular surface, enabling controlled release over an extended period. This controlled-release mechanism enhances drug efficacy while reducing the frequency of administration [166,167]. Additionally, HA hydrogels can be incorporated into contact lenses to improve comfort and alleviate dryness experienced by contact lens wearers. The hydrogel’s capability to retain water helps prevent corneal dehydration and enhances the overall lens-wearing experience [48]. HA, being a natural component of tear film, plays a vital role in maintaining ocular surface health. Therefore, HA hydrogels can serve as artificial tear substitutes by providing lubrication and retaining moisture on the ocular surface, offering relief to individuals with dry eye syndrome [167,168]. Ongoing research and development in the realm of HA hydrogels for ophthalmology strive to explore new applications and optimize their properties to achieve improved clinical outcomes. It is important to note that the specific utilization of HA hydrogels may vary based on individual patient conditions and the intended therapeutic objectives.

### 4.5. Three-Dimensional Bioprinting

**Figure 21:**
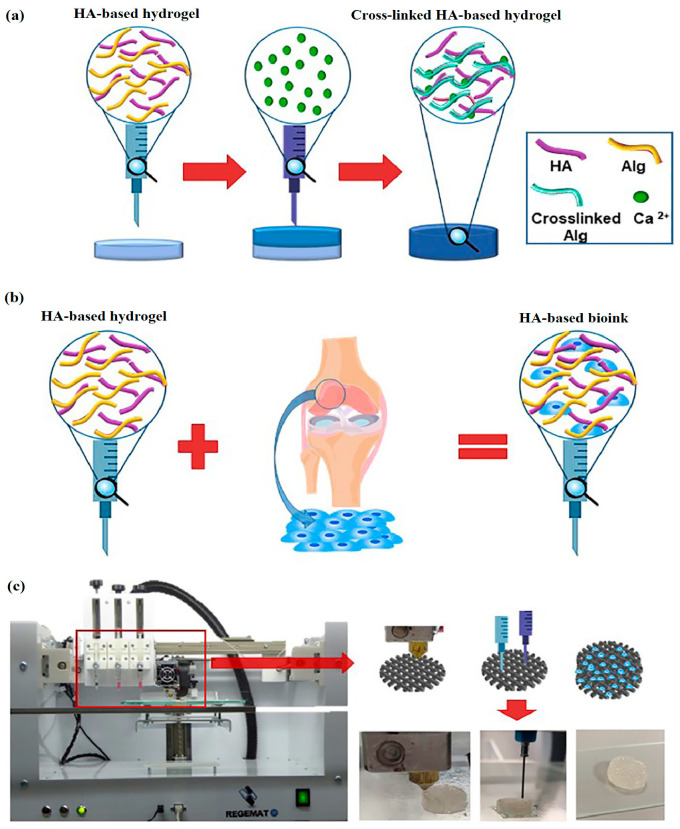
(**a**) A formulation of hydrogel; (**b**) creating a bioink (hydrogel) based on HA; (**c**) illustration depicting the process of 3D bioprinting for articular cartilage engineering. Reprinted with permission from ref. [172]. 2020, Elsevier.

In the world of 3D bioprinting, HA hydrogels play a vital role. They can be filled with cells, growth factors, and bioactive molecules, enabling the creation of intricate living tissues and organ structures. The precision offered through 3D bioprinting allows for the meticulous placement of cells and biomaterials, resulting in complex tissue architectures that closely resemble natural tissue environments [169]. Using HA hydrogel in 3D bioprinting offers numerous advantages: it promotes cell adhesion, proliferation, and migration; enhances mechanical properties; and boosts the overall functionality of printed constructs. Furthermore, HA is a naturally occurring substance in the body, which lowers the chances of immune rejection or adverse reactions when employed in tissue engineering [170,171,172]. The utilization of HA hydrogels in 3D bioprinting holds great promise in fields like tissue engineering, regenerative medicine, drug screening, and disease modeling. Researchers are actively seeking ways to enhance the mechanical properties, degradation kinetics, and bioactivity of HA hydrogels to mimic native tissue properties better and improve bioprinted construct functionality. Antich et al. highlighted the potential of HA hydrogel as a sought-after biomaterial for engineering articular cartilage using 3D bioprinting as depicted in Figure 21 [172].

### 4.6. Three-Dimensional Culture and Disease Modeling

HA hydrogels offer a 3D environment that more closely mimics the natural extracellular surrounding matrix compared to conventional two-dimensional cell tradition schemes. This three-dimensional formation permits cells to communicate with one another and their conditions in a more physiologically related way. Researchers leverage HA hydrogels to develop three-dimensional cell tradition models for examining cell behavior, tissue formation, drug response, and disease progression [173,174]. These models are important for comprehending intricate biological processes and for drug-screening applications. Also, HA hydrogels are utilized to cultivate disease models that replicate the micro-setting of various illnesses, such as cancer, tissue scarring, and inflammatory disorders. By incorporating distinct cell types, growth factors, and other bioactive molecules within the HA hydrogel matrix, scientists can simulate facets of the pathology of disease in a controlled laboratory setting [175,176]. Disease models founded on HA hydrogels are valuable tools for studying disease mechanisms, testing potential therapies, and customized medical approaches.

## 5. Conclusions and Prospects

While HA hydrogels present endless customization options and have proven useful in various biomedical applications, hurdles remain in their production and analysis. The creation of HA hydrogels has emerged as an exciting avenue in biomaterials, offering tailor-made possibilities for a wide range of biomedical uses. These gels can be customized to fulfill precise needs in tissue engineering, drug delivery, and wound healing, thanks to their adjustable traits like strength, swelling, and breakdown speed. Different techniques, from chemical cross-linking to physical gelation, can be utilized to craft HA hydrogels, adding flexibility to their manufacture. Yet, making HA hydrogels frequently involves numerous intricate steps, making the production process inherently demanding. This might be time-consuming and demand specialized knowledge. Variability between batches is a concern as gel properties can fluctuate based on factors such as the source of HA, synthesis conditions, and cross-linking agents employed. This presents difficulties in maintaining uniformity and reliability. Characterizing HA hydrogels is also challenging, as each analysis approach has constraints and may not entirely capture the intricacies of gel properties. Achieving ideal mechanical traits such as long-term stability, precise degradation rates, and understanding cell/tissue interactions continue to be obstacles affecting effectiveness. Although HA boasts biocompatibility, degradability, and moisture retention, optimizing stability and degradation speed is crucial for ensuring prolonged safety in vivo. Moving forward, there is enormous potential for the creation of innovative synthesis methods and characterization techniques to tackle these limitations.

## Figures and Tables

**Table 1:** Cross-Linking Methods for Forming HA Hydrogels: A Comparative Summary.

Cross-Linking Method	Reagents/Conditions Used	Applications	References
Carbodiimide cross-linking	EDC	Tissue engineering, drug delivery	[22]
Diisocyanate cross-linking	HDI, bis (β- isocyanatoethyl) disulp hide	Tissue engineering, wound healing, drug delivery	[23]
Michael addition	Thiol groups (cysteine, DTT), -VS, -MAL, -AC	Tissue engineering, drug delivery, controlled drug/gene release	[24,25,26,27,28]
Esterification	EDC/HOBt	Drug delivery, wound healing, tissue engineering	[29,30]
Diels–Alder reaction	Norbornene, tetrazine, furan, maleimide	Injectable hydrogels, photo-degradable hydrogels, controlled drug release	[31,32]
Photo cross-linking	Photo-initiator, UV, or visible light	Tissue engineering, wound healing, controlled drug release	[33,34]
Thiol-ene click	(Meth)acrylate and thiol functional groups without initiators under physiological conditions	cell culture, contact lenses	[35,36]
Ether reaction	BDDE; DVS under room temperature conditions	Drug delivery	[36]
Amidation	EDC, CMPI, CDMT	Drug delivery	[35,36]
Hydrazone linkage	Hyaluronic acid Adipic acid dihydrazide (HA-ADH) reacted with aldehydes or ketones	Drug delivery	[37,38,39]
Temperature-induced gelation	Thermo-responsive polymers (PNIPAAm)	Injectable hydrogels, tissue engineering	[40,41]
Covalent augmentation	PEGDA	Enhanced mechanical properties, controlled drug delivery	[42]
Freeze–thawing	Repeated freezing and thawing	Porous structure, controlled drug release	[43,44]
Enzymatic cross-linking	Horseradish peroxidase, tyramine	Tissue engineering, drug delivery, wound healing	[45,46]

**Table 2:** Applications of HA in Ophthalmology.

Ophthalmology Application	Target	HA Function
Artificial tear and eye drops	Ocular surface	1. Increase the moisture retention [99] 2. Better tear film stability, ocular surface regularity, and quantity of conjunctival goblet cells [166]
Tissue engineering	Corneal	Benefit of cell growth and wound healing [163]
In situ gel	Ocular surface	1. Help the drug absorption and drug delivery [47] 2. Adjust the viscosity and degradation time [48]

## Data Availability

Not applicable.
